# Behavior Differences Between Search-and-Rescue and Pet Dogs

**DOI:** 10.3389/fvets.2018.00118

**Published:** 2018-06-05

**Authors:** Elizabeth Hare, Kathleen M. Kelsey, James A. Serpell, Cynthia M. Otto

**Affiliations:** ^1^Penn Vet Working Dog Center, School of Veterinary Medicine, University of Pennsylvania, Philadelphia, PA, United States; ^2^Dog Genetics, LLC, Sunnyside, NY, United States; ^3^Department of Clinical Sciences & Advanced Medicine, School of Veterinary Medicine, University of Pennsylvania, Philadelphia, PA, United States

**Keywords:** dog, behavior, questionnaire, working dog, trainability, fear, aggression, search and rescue

## Abstract

Behavioral traits such as trainability, fearlessness, and energy are required for dogs to succeed as search-and-rescue (SAR) dogs. Certification by the Federal Emergency Management Agency (FEMA) ensures that dogs and handlers have extensive training and have demonstrated specific skills in the field. To determine whether behavioral differences exist between SAR and pet dogs, and between FEMA-certified USAR and non-FEMA-certified SAR dogs, the Canine Behavioral Assessment and Research Questionnaire (C-BARQ) was administered to 129 SAR dogs participating in the post-9/11 medical surveillance study and a breed-matched sample of 2,131 pet dogs. Non-parametric mixed models were fit for each C-BARQ subscale with explanatory variables SAR/non-SAR status, FEMA certification status, breed, sex, neuter status, and age. SAR dogs had higher scores for trainability (*P* < 0.001) and energy (*P* < 0.001), and lower scores for aggression toward strangers (*P* < 0.01), aggression and fear toward dogs (*P* < 0.01), fear of dogs (*P* < 0.001), chasing (*P* < 0.001), fear of strangers (*P* < 0.001), and non-social fear (*P* < 0.001) than pet dogs. FEMA-certification was associated with lower fear of dogs (*P* < 0.05) and separation-related issues (*P* < 0.01) than non-FEMA certified SAR dogs. The traits identified in this study could provide guidance for more efficient selection of candidate SAR dogs and breeding stock.

## Introduction

Search-and-rescue (SAR) and human remains detector (HRD) dogs are selected and trained for behaviors correlated with success in the field. The United States Federal Emergency Management Agency (FEMA) certification includes “proper command control, agility skills, a focused bark alert to indicate a live find, and a willingness to persist to search for live victims in spite of possible extreme temperatures and animal, food and noise distractions. The canine must also be confident enough to search independently and must be able to negotiate slippery surfaces, balance wobbly objects underneath his feet and go through dark tunnels^1^.” A survey of search dog handlers in the UK identified seven priority behaviors: acuity of sense of smell, incentive to find a hidden object, tendency to hunt by smell alone, ability to learn from being rewarded, tendency not to be distracted when searching, consistency of behavior from day to day, and motivation to chase an object (1). In the US, behaviors thought to be associated with successful search work are prey drive, hunt drive, and ball drive (2).

Research using behavior questionnaires to study working dogs has been primarily focused on guide and service dogs. The C-BARQ (Canine Behavioral Assessment and Research Questionnaire) is a questionnaire completed by a dog's owner or caretaker. Most of the individual items are grouped into subscales describing a more broad behavioral trait, such as trainability, owner-directed aggression, stranger-directed aggression, rivalry or chasing. A prototype of the C-BARQ was validated in a population of 1,067 Seeing Eye dogs. Puppy-raiser evaluations on the behavior subscales: stranger fear, stranger aggression, non-social fear, owner aggression, dog fear/aggression, and trainability at 12 months of age were predictive of behavioral reasons dogs were released from the training program several months later (3). In a larger, related study of 7,696 dogs from five guide and service dog programs, C-BARQ scores at 6 and 12 months of age for 27 out of a possible 36 temperament traits were significantly different between dogs who successfully completed training and those released for behavioral reasons (4). In a sample of potential military working dogs, high scores on C-BARQ for trainability at 12 months were associated with better performance on standardized behavior test at 17 months, and negatively associated with non-social and stranger-directed fear (5).

After the terrorist attacks of September 11, 2001, between 250 and 300 dogs deployed to the World Trade Center, Fresh Kills Landfill, and the Pentagon (6). The health and behavior of these dogs was under surveillance until 2016, when the final dog responding to the attacks died (6–8). The handlers of the dogs that deployed after these attacks, along with handlers of SAR or HRD dogs who did not deploy to that event completed the C-BARQ. For the present study, C-BARQ test items and scores from pet dogs were also analyzed. The goal of the study was to determine whether behavior differences exist between SAR dogs whose handlers completed the C-BARQ within 1 year after the 9/11 deployment and pet dogs whose owners completed the C-BARQ from May 2005 through May 2010. A secondary goal was to ask whether SAR dogs who had completed training and FEMA certification had different behavior scores than SAR dogs who had not completed FEMA certification.

## Materials and methods

### Participants

The C-BARQ questionnaire (4, 9) was administered annually to the handlers of 129 SAR dogs as part of their participation in a medical surveillance study of SAR dogs who were either deployed to the World Trade Center, Pentagon, or Staten Island Landfill or served as control SAR dogs (not deployed after the attacks) in the study (6–8). The study year 1 (from September 11, 2001 to September 10, 2002) questionnaire results were utilized in this study.

The C-BARQ questionnaire was also administered to owners of 2,131 pet dogs. Pet dogs were solicited through one of two methods. They either received a mailing because they were clients of the Veterinary Hospital of the University of Pennsylvania or the completed the questionnaire via an online survey that was advertised via an article in the newsmagazine of the Veterinary Hospital of the University of Pennsylvania, USA (http://www.vet.upenn.edu/bellwether/v64/article10.shtml) and by notices sent to Philadelphia-area veterinary clinics and the top 20 USA breed clubs based on AKC registrations. Availability of the survey then spread via word of mouth. Pet dogs were included if their breed was represented in the sample of SAR dogs (Table 1). The entire population eligible to be included in this study consisted of 1,179 males (938 neutered and 241 intact) and 1,081 females (916 neutered and 165 intact). C-BARQs were completed between May 2005 and May 2010.

**Table 1:** Breed distribution of dogs in 9/11 surveillance study and pet dogs.

**Breed**	**SAR dogs**	**Pet dogs**
Airedale Terrier	2	39
Australian Cattle Dog	2	105
Australian Shepherd	4	151
Belgian Malinois	1	26
Border Collie	8	152
Doberman Pinscher	1	135
English Springer Spaniel	1	53
German Shepherd Dog	53	381
German Shorthaired Pointer	1	26
Golden Retriever	13	285
Labrador Retriever	40	612
Rottweiler	3	166

For the SAR dog population, 129 completed CBARQs were included. The majority of dogs were deployed to 9/11 (*n* = 86) whereas 46 SAR dogs were not deployed to 9/11. Eighty-one SAR dogs were FEMA certified or eligible (USAR) and 48 SAR dogs were not affiliated with FEMA. There were 74 male dogs, of which 74% were neutered and 55 female dogs of which 93% were neutered. The median age for SAR dogs was 4 years with a range from 1 to 11. Age was rounded to the nearest whole number in years for further calculations. The entire pet and SAR population consisted of 1,179 males of which 80% were neutered and 1,081 females of which 85% were neutered. The median age was 3 years with a range from 1 to 20 years for both the pet and SAR dogs.

The CBARQ study was approved by the University of Pennsylvania Institutional Animal Care and Use Committee and was exempt from Institutional Review Board approval because personal information was not collected about the dog owners.

### Statistical analysis

Behavior subscales were computed as described in Hsu and Serpell (9). Descriptive statistics are shown in Table 2. Cronbach's alpha, a measure of the agreement of the items within each subscale, was computed using the Cronbach function in the “psy” package (10) in the R statistical software package [(11); open source software available at https://www.r-project.org]. Alpha varied from 0.48 to 0.87, with most subscales above 0.70, indicating good agreement between items. The distributions of all subscales failed the Shapiro-Wilk test for normality. Several transformation functions were attempted, however, all the scores except for trainability had positive skewness with many values near 0 and few high values and there was no transformation that made the distributions more normal.

**Table 2:** Descriptive statistics for C-BARQ subscales.

	**All Sar dogs**					**Non-FEMA SAR dogs**		**FEMA SAR dogs**	
**Subscale**	**Mean**	***SD***	**Skewness**	**Cronbach's Alpha**	**Number of Items**	**Mean**	***SD***	**Mean**	***SD***
Trainability	3.28	0.34	−0.42	0.48	8	3.26	0.35	3.29	0.33
Aggression toward strangers	0.35	0.39	1.72	0.83	10	0.50	0.49	0.27	0.30
Aggression toward owner	0.04	0.16	4.64	0.78	8	0.08	0.25	0.02	0.07
Fear and aggression toward dogs	0.64	0.53	1.31	0.81	8	0.74	0.62	0.59	0.47
Aggression toward dogs	0.91	0.79	1.12	0.87	4	1.04	0.90	0.85	0.71
Fear of dogs	0.39	0.59	1.63	0.87	4	0.51	0.71	0.32	0.50
Dog rivalry	0.53	0.57	2.10	0.74	4	0.70	0.68	0.34	0.47
Chasing	1.25	1.00	−0.21	0.84	4	1.49		1.11	0.89
Fear of strangers	0.12	0.30	2.01	0.82	4	0.18	0.36	0.08	0.25
Non-social fear	0.32	0.38	1.47	0.65	6	0.44	0.47	0.25	0.29
Separation problems	0.30	0.43	1.71	0.78	8	0.43	0.51	0.22	0.36
Touch sensitivity	0.47	0.59	1.61	0.49	4	0.59	0.65	0.40	0.55
Excitability	2.08	0.68	0.003	0.78	6	2.14	0.70	2.04	0.68
Attachment/Attention-seeking	2.00	0.63	0.14	0.65	6	2.23	0.69	1.86	0.55
Energy	2.61	0.81	−0.18	0.74	2	2.55	0.81	2.65	0.81

In order to determine whether SAR dogs differed from pet dogs on behavior subscales, and whether there were further differences associated with FEMA certification, non-parametric wmodels were fit to each subscale using the “np” package (12) in R. Non-parametric methods are used when a dependent variable is not normally distributed, and this R package fits models to ordinal dependent variables such as C-BARQ subscales. In addition to SAR and FEMA status, explanatory variables included breed, sex, neuter status, and age. Models were fit using a backward elimination strategy using the “drop1” R function. The first, full model for each subscale contained all explanatory variables. Subsequent refined models contained only variables that were significant at the *P* < 0.05 level. This process resulted in two steps and models for most of the subscales except fear of dogs and separation problems, which required three models. Because differences between means cannot be tested directly using non-parametric models, partial regressions were carried out using the “np” package's “npplot” function to determine the estimated mean values for each category when SAR status and/or FEMA status was found to be a significant factor.

## Results

The final model for each C-BARQ subscale is presented in Table 3. *P*-values are given for any explanatory variable that was significant at the 0.05 level. Means for SAR and pets, as well as FEMA and non-FEMA certified SAR dogs are provided. SAR dogs had higher scores for trainability (*P* < 0.001) and energy (*P* < 0.001), and lower scores for aggression toward strangers (*P* < 0.01), aggression and fear toward dogs (*P* < 0.01), fear of dogs (*P* < 0.001), chasing (*P* < 0.001), fear of strangers (*P* < 0.001), and non-social fear (*P* < 0.001) than pet dogs. FEMA-certification was associated with lower fear of dogs (*P* < 0.05) and separation-related problems (*P* < 0.01) than non-FEMA certified SAR dogs.

**Table 3:** Final models for C-BARQ subscales (*NS* = not significant at *P* < 0.05 in previous model in backward elimination).

**Trait**	**Model R^2^**	**SAR**	**SAR mean**	**non-SAR Mean**	**FEMA**	**FEMA mean**	**non-FEMA mean**	**Breed**	**Age**	**Sex**	**Neutered**
Trainability	0.08	<2.2e-16	3.16	2.64	NS	NA	NA	0.003	<2.2e-16	NS	NS
Aggression toward strangers	0.12	0.01	0.41	0.47	NS	NA	NA	<2.2e-16	0.003	NS	NS
Aggression toward owner	0.02	NS	NA	NA	NS	NS	NA	<2.2e-16	NS	NS	NS
Aggression and Fear of dogs	0.10	0.01	0.69	0.76	NS	NA	NA	<2e-16	<2e-16	NS	NS
Aggression toward dogs	0.13	NS	NA	NA	NS	NA	NS	<2.2e-16	<2.2e-16	NS	NS
Fear of dogs	0.02	<2.2e-16	0.53	0.63	0.04	0.62	0.63	0.03	NS	0.08	NS
Do rivalry	0.06	NS	NA	NA	NS	NA	NA	<2.2e-16	0.003	NA	NS
Chasing	0.15	<2e-16	1.24	1.96	NS	NA	NA	<2e-16	0.04	<2e-16	NS
Fear of strangers	0.09	<2e-16	0.29	0.36	NS	NA	NA	<2e-16	0.03	<2e-16	NS
Non-social fear	0.05	<2e-16	0.39	0.73	NS	NA	NA	0.01	NS	NS	0.02
Separation problems	0.02	NS	NA	NA	0.005	0.3	0.49	NS	<2e-16	NS	<2.2e-16
Touch sensitivity	0.04	NS	NA	NA	NS	NA	NA	NS	<2.2e-16	0.005	0.02
Excitability	0.03	NS	NA	NA	NS	NA	NA	NS	<2.2e-16	NS	NS
Attachment/Attention-seeking	0.05	NS	NA	NA	NS	NA	NA	<2e-16	0.01	NS	NS
Energy	0.17	<2.2e-16	2.79	2.23	NS	NA	NA	NS	<2.2e-16	NS	NS

## Discussion

This is the first study comparing behavior traits measured by the C-BARQ in working SAR dogs and pet dogs. There have been analyses of behavior in puppies with the goal of using behavior measures to select dogs for work early in life. In a study of Swedish military working German Shepherd Dogs comparing C-BARQ scores with the outcome of a temperament test for acceptance into the program, trainability was significantly higher in dogs that passed the test, and stranger-directed aggression, stranger-directed fear, and non-social fear were significantly lower in dogs who passed the screening test (5).

In a study of guide and service dog puppies, using a logistic regression model with successful training as the dependent variable and C-BARQ scores at 6 months as explanatory variables, 27 of the C-BARQ traits explained significant proportions of the variation in success (4). Many of these traits from a 6-month C-BARQ were the same as those associated with working dog status in the present study, including trainability, stranger-directed aggression, owner-directed aggression, dog-directed aggression, non-social fear, stranger-directed fear, and chasing. The present study did not find differences in touch sensitivity, separation problems, or excitability between SAR and pet dogs. These traits might be more important for guide dog work than for SAR work since guide dogs work in closer proximity to humans where touch sensitivity is more problematic, and guide dogs in training are not required to be alone frequently. The guide and service dog study found significant negative associations with success for dog-directed aggression, rivalry, and attachment/attention-seeking, while the present study does not. The same model was fitted with C-BARQ scores from puppies at 12 months of age. Trainability was significantly higher in successful dogs, and all other behavior characteristics measured in the present study had negative relationships with success. Characteristics that did not distinguish pets from SAR dogs in the present study but did have a relationship with success in the guide and service dog study were dog-directed aggression, dog rivalry, and attachment/attention-seeking. The relationships between C-BARQ behavior traits and successful training as a service dog were similar at both ages, suggesting that it may be possible to use some C-BARQ subscales to screen and select dogs for SAR work as early as 6 months.

Boldness was found to be associated with high performance in working dog tests in Swedish female German Shepherd Dogs and Belgian Tervurens (13). The Dog Mentality Assessment is a broad-ranging test of a dog's aptitudes and differs substantially from the C-BARQ in that is not a questionnaire completed by owners but a behavior test scored by a judge. However, Svartberg (14) found correlations between the Dog Mentality Assessment boldness measures and C-BARQ fear subscales. High performing dogs had higher boldness scores then low performing dogs in agreement with the present findings that several types of fear (fear of dogs, fear of strangers, and non-social fear) are negatively associated with working dog status.

The only behavior differences between FEMA-certified USAR dogs and uncertified SAR dogs were lower fear of dogs and separation-related problems. This could be related to a general lack of fear that seems to be associated with successful working dogs, and could be a result of training. SAR training involves frequent travel to training events with other dogs, and dogs are required to work at a greater distance from their handlers than guide or service dogs.

The present study differs from the other behavior studies discussed here because it utilized two different populations of people to respond to the C-BARQ. It is unknown whether and how the increased knowledge of canine behavior possessed by working dog handlers relative to pet dog owners affects their understanding of the terminology of the C-BARQ or their ability to assess their dogs. Thus, the differences in subscales reported here could be biased upward or downward.

It is not clear whether the behavior differences found in the present study are due to selection of dogs with these traits or whether they result from training. Future research at a facility such as the Penn Vet Working Dog Center where puppy behavior is tracked during development could provide a means of observing changes in behavior during development and comparing dogs with different levels of success in SAR work. Future work should be aimed at developing questionnaires that focus on the specific requirements for SAR dogs such as the ability to work independently at a distance from the handler, persistence on odor, and ability to learn odors. A specialized temperament test involving such traits would facilitate the identification of individual dogs with potential to be trained as odor detection dogs.

Our results can be used to inform the selection of puppies and juvenile dogs for training as SAR dogs. More efficient selection would result in reduced costs associated with the purchase and training of dogs that are less likely to successfully complete FEMA certification. Some of the C-BARQ subscales for fear and aggression have been associated with specific genomic regions (15) and others such as trainability and aggression have been found to be heritable (16), so these findings can also be applied in selective breeding programs to produce future SAR dogs.

## Author contributions

EH conducted statistical analysis. KK collected SAR dog data. JS provided interpretation of C-BARQ findings and petdog data, and CO directed the research. All authors reviewed and edited the manuscript.

### Conflict of interest statement

EH is the sole proprietor of Dog Genetics LLC, which provides statistical and genetic analysis for working dog organizations. The other authors declare that the research was conducted in the absence of any commercial or financial relationships that could be construed as a potential conflict of interest.

## Footnotes

^1^https://www.fema.gov/canine-handler-certification
